# Meta-Analysis of the Influence of Taijiquan Exercise on the Disease Severity and Balance Function of Patients with Mild to Moderate Parkinson’s Disease Under Different Control Measures

**DOI:** 10.1093/geroni/igaf122.1832

**Published:** 2025-12-31

**Authors:** Yan Zhang, Yunhua Cao

**Affiliations:** North China Electric Power University, Beijing, Beijing, China (People’s Republic); North China Electric Power University, Beijing, Beijing, China (People’s Republic)

## Abstract

**Objective:**

To explore the influence of Taijiquan exercise on the disease severity and balance function of patients with mild to moderate Parkinson’s disease under different control interventions.

**Method:**

As of February 15, 2025, a literature search was conducted in four databases, including PubMed. The inclusion and exclusion criteria for the literature were formulated, and a literature information form was created. Two experienced evaluators independently completed the literature screening and data extraction. Stata18.0 software was used for data processing such as heterogeneity testing and subgroup analysis.

**Results:**

A total of 15 randomized controlled trials were included, with a total of 17 groups of research data and 784 participants. The results showed that the combined intervention of different control measures and Taijiquan had a significant difference in the improvement of disease severity as a whole ([WMD = -3.804, 95%CI(-5.345, -2.263), P < 0.001]). At the same time, there was a significant difference in the improvement of the balance function of patients with mild to moderate Parkinson’s disease compared with various control measures ([WMD = -3.498, 95%CI(-5.096, -1.901), P < 0.001]).

**Conclusion:**

Taijiquan exercise intervention can significantly improve the disease severity and balance function of patients with mild to moderate Parkinson’s disease. It is recommended that patients with mild to moderate Parkinson’s disease actively engage in Taijiquan exercise. Since the degrees of influence of various interventions (such as drugs, routine nursing in the department of neurology, etc.) combined with Taijiquan exercise vary, more in-depth research is still needed on the impact of Taijiquan intervention on Parkinson’s disease.

